# The value of machine learning models in predicting lung metastasis in papillary thyroid carcinoma

**DOI:** 10.3389/fendo.2026.1776463

**Published:** 2026-09-11

**Authors:** Haiqing Zhu, Wei Tan, Yuanqi Zheng, Jianyi Liu, Xiaowei Peng, Jing Peng, Zhenyu Zou, Zongxin Liu, Xinghan Zhou, Feng Shi

**Affiliations:** 1 Department of Nuclear Medicine, The Affiliated Cancer Hospital of Xiangya School of Medicine, Central South University/Hunan Cancer Hospital, Changsha, Hunan, China; 2 Graduate Collaborative Training Base of Hunan Cancer Hospital, Hengyang Medical School, University of South China, Henyang, Hunan, China; 3 Department of Thyroid Surgery, The Affiliated Cancer Hospital of Xiangya School of Medicine, Central South University/Hunan Cancer Hospital, Changsha, Hunan, China

**Keywords:** LASSO, LR, lung metastasis, machine learning, nomogram, papillary thyroid carcinoma, prediction model

## Abstract

**Objective:**

To construct a machine learning (ML) model to predict the risk of lung metastasis in papillary thyroid carcinoma (PTC).

**Methods:**

This retrospective study included 681 PTC patients who received radioactive iodine (RAI) treatment for the first time. Among them, 71 patients were diagnosed with lung metastasis (10.4%). The patients’ age, sex, stimulated thyroglobulin (s-Tg) level ahead of ^131^I treatment, unilateral or bilateral lobe involvement, maximum tumor diameter, number of invading tumors (extracted from postoperative pathological reports, defined as the number of structures involved by extrathyroidal extension), as well as T and N status, were evaluated. The dataset was randomly divided into a training set and a testing set in a 7:3 ratio, and least absolute shrinkage and selection operator (LASSO) regression was used for feature screening. Logistic Regression (LR), Random Forest (RF), Support Vector Machine (SVM) and extreme gradient boosting (XGBoost) were constructed, and we used 10-fold cross-validation in the validation of the models, assessed through the receiver operating characteristic (ROC) curves, calibration curves, area under the precision recall curve (AUPRC) and decision curve analysis (DCA).

**Results:**

The selected features in the final model included “maximum tumor diameter”, “number of invading tumors”, and “s-Tg”. The RF model had the highest area under the curve (AUC) of 0.935, whereas the SVM had the lowest generalization ability (AUC = 0.863). In the testing of all models, LR achieved an AUC of 0.928. Although the RF model had a slightly higher AUC of 0.935, the LR model was ultimately chosen due to its superior generalization ability as indicated by its highest performance in AUPRC of 0.74 in cross-validation. DCA showed that under most threshold probabilities, the net benefit of the LR model exceeded other models. A nomogram was used to display the LR results.

**Conclusion:**

LASSO regression analysis identified that large primary tumor, high number of invading tumors, high s-Tg level ahead of ^131^I treatment might be considered as risk factors for lung metastasis in PTC patients. Among the ML models constructed accordingly, the LR model demonstrated strong performance in predicting lung metastasis of PTC.

## 1 Introduction

Papillary thyroid cancer(PTC) is the most common type of thyroid cancer, accounting for approximately 85% to 90% of all thyroid cancers (1). The long-term prognostic profile of PTC is generally positive, featuring excellent survival rates: 96% at the 5-year mark, 93% at 10 years, and more than 90% when followed for 20 years (2). However, the prognosis deteriorates significantly when distant metastasis occurs (2–4), and the most common site of distant metastasis is the lung (5). Early identification of patients at risk for lung metastasis is therefore critical, as it enables timely risk stratification and appropriate treatment planning. For patients with lung metastasis, ^131^I treatment remains the first-line option (6). Studies have shown that if a whole-body ^131^I scan (RxWBS) indicates the development of metastatic lesions after treatment, the 5-year survival rate is 87%, and the 10-year survival rate is 69.2% (7). Among them, ^131^I treatment can achieve a high complete remission rate for diffuse small metastatic lesions. However, current methods of determining the appropriate dosage for ^131^I treatment, particularly fixed-dose and empirical methods, are based on limited clinical evaluation, leading to inconsistent treatment efficacy and potential recurrence risks (8–12). Therefore, more precise prediction methods for lung metastasis in PTC are imperative to improve patient risk stratification and inform clinical decision-making, including the assessment for appropriate postoperative management.

The applicability of machine learning (ML) to predict diagnoses and clinical outcomes has previously been demonstrated (13). Compared with traditional statistical analysis methods, ML is better at analyzing complex data interactions and nonlinear relationships, especially in complex situations where multiple interdependent factors need to be considered (14). Current methods for predicting lung metastasis in PTC patients often rely on traditional clinical evaluation, which can miss crucial patterns in complex datasets. ML methods, on the other hand, can uncover these patterns by analyzing large volumes of data and incorporating diverse risk factors.

In this study, we performed a retrospective analysis of 681 patients with PTC who underwent their first course of radioactive iodine (RAI) therapy, incorporating baseline demographic data, clinical features, and laboratory findings. The dataset was integrated to enable a comprehensive analysis of risk factors associated with lung metastasis in patients with PTC. Therefore, this study aimed to construct an ML-based predictive model and develop a complementary nomogram to facilitate early identification of lung metastasis risk. This approach may support timely detection and inform the formulation of more individualized treatment strategies.

## 2 Methods

### 2.1 Patients selection

This study included PTC patients who received RAI treatment for the first time in the Department of Nuclear Therapy at Xiangya Medical College Affiliated Cancer Hospital of Central South University from January 2017 to June 2020. The inclusion criteria were as follows: 1) Postoperative pathology confirmed papillary thyroid carcinoma; 2) Complete baseline and follow-up data of subjects, including laboratory tests, imaging examinations, and clinical diagnosis and treatment records. Patients were excluded if any of the following criteria were met: 1) negative suppressed thyroglobulin (Tg) (<1 ng/ml); 2) Complicated with second primary tumor, or concurrent with other pathological types of thyroid carcinoma (e.g., medullary carcinoma, anaplastic carcinoma,etc.); 3) Follow-up duration less than 2 years or incomplete follow-up data (to avoid loss to follow-up). Finally, 681 eligible PTC patients (449 females and 232 males) were included in this study, 71 of whom were diagnosed with pulmonary metastasis. PTC patients who met any one of the following criteria were diagnosed with lung metastasis (7, 15): 1) pathological examination confirming that the metastatic lesion in the lung originated from thyroid tissue; 2) chest computed tomography (CT) revealing pulmonary nodules, with RxWBs combined with Single-Photon Emission Computed Tomography/Computed Tomography (SPECT/CT) demonstrating positive ¹³¹I uptake in the lungs accompanied by elevated serum Tg levels; 3) elevated serum Tg or thyroglobulin antibody (TgAb) levels with chest CT suggesting pulmonary nodules, but negative pulmonary ¹³¹I uptake on RxWBs combined with SPECT/CT imaging; 4) elevated serum Tg or TgAb levels showing positive pulmonary ¹³¹I uptake on RxWBs combined with SPECT/CT imaging, but without identifiable pulmonary nodules on chest CT.

### 2.2 Research variables

The following clinical information was collected from each patient: age, sex, maximum tumor diameter, number of invading tumors defined as the number of structures involved by extrathyroidal extension (ETE) based on postoperative pathological reports, including muscle, blood vessels, nerves, and trachea/esophageal wall (0 = no ETE; 1–4 = number of involved structures), unilateral or bilateral lobe involvement, T status, N status (staged by postoperative pathology), stimulated thyroglobulin (s-Tg) level before RAI treatment, and thyroglobulin antibody (TgAb) level before RAI treatment. s-Tg is defined as the Tg level measured when thyroid-stimulating hormone (TSH) > 30 mIU/L before receiving RAI treatment. After initial variable screening, TgAb was not retained in the final model. To select and filter the most relevant predictors, we used the least absolute shrinkage and selection operator (LASSO) regression.

### 2.3 Research design and statistical analysis

All statistical analyses and data visualizations were conducted using R 4.4.1 and Python 3.13. Continuous data are expressed as means ± standard deviations or medians (Q1, Q3), whereas categorical data are expressed as frequencies (percentages). Intergroup comparisons were evaluated using chi-square tests for categorical variables and independent sample t-tests or nonparametric tests for continuous variables. Of the 681 patients, 71 (10.4%) had lung metastases and 610 (89.6%) did not, indicating a class imbalance.

Prior to model construction, continuous variables were standardized using Z-score normalization to ensure consistent feature scaling. All preprocessing steps were performed on the training set and then applied to the validation set to prevent data leakage.

All patients were randomly divided into a training set and a validation set at a 7:3 ratio by means of the caret package. Four ML models, namely, Logistic Regression (LR), Random Forest (RF), Support Vector Machine (SVM), and extreme gradient boosting (XGBoost), were constructed using the LASSO-selected features. Hyperparameter tuning for each model was performed using grid search. Ten-fold cross-validation was conducted within the training set to evaluate model performance. Model performance was further assessed using ROC curves and area under the precision-recall curve (AUPRC). Given the imbalanced nature of the dataset, AUPRC was considered alongside ROC analysis (16). Calibration curves and decision curve analysis (DCA) were also used to assess model performance. The LR model results were visualized using a nomogram, which provides a comprehensive evaluation of disease prediction by integrating multiple clinical predictors (17).

## 3 Results

### 3.1 Basic characteristics of patients

Among the 681 PTC patients who received RAI treatment for the first time included in the study, 71 were diagnosed with lung metastasis after follow-up, with a metastasis rate of 10.4%. A summary of patient characteristics is presented in **Table 1**. Among these variables, unilateral or bilateral lobe involvement (p<0.05), maximum tumor diameter (p<0.05), number of invading tumors (p<0.05), T (p<0.05), and s-Tg (p<0.05) were significantly different between the two groups; however, there were no significant differences in sex, age, or N between the two groups of patients (p>0.05).

**Table 1:** Comparison of basic information between the lung metastasis group and the non-lung metastasis group.

Variables	Total (n = 681)	Non-lung metastasis group (n = 610)	Lung metastasis group (n = 71)	p
Sex, n (%)				0.655
Female	449 (66)	400 (66)	49 (69)	
Male	232 (34)	210 (34)	22 (31)	
Age, median (Q1, Q3)	39 (31, 48)	39 (31, 48)	39 (28, 53.5)	0.798
Unilateral or bilateral lobe involvement^a^, n (%)				0.008
Unilateral	375 (55)	347 (57)	28 (39)	
Bilateral	306 (45)	263 (43)	43 (61)	
Maximum tumor diameter^a^, median (Q1, Q3)	1.7 (1.1, 2.8)	1.5 (1, 2.5)	3 (2.1, 4)	< 0.001
Number of invading tumors^a^, n (%)				< 0.001
0	499 (73)	472 (77)	27 (38)	
1	148 (22)	127 (21)	21 (30)	
2	20 (3)	2 (0)	18 (25)	
3	11 (2)	6 (1)	5 (7)	
4	3 (0)	3 (0)	0 (0)	
T^b^ stage, n (%)				< 0.001
1	288 (42)	282 (46)	6 (8)	
2	118 (17)	106 (17)	12 (17)	
3	162 (24)	140 (23)	22 (31)	
4	113 (17)	82 (13)	31 (44)	
N^b^ stage, n (%)				0.271
0	38 (6)	32 (5)	6 (8)	
1	643 (94)	578 (95)	65 (92)	
s-Tg, median (Q1, Q3)	9.03 (3.15, 29.36)	7.68 (2.9, 20.24)	283.2 (42.51, 489)	< 0.001

Group comparisons were performed using chi-square tests (categorical variables) and t-tests or non-parametric tests (continuous variables). *p* value < 0.05 was considered statistically significant.

^a^Variables directly extracted from postoperative pathological reports (e.g., Maximum tumor diameter).

^b^Variables derived from postoperative pathology according to TNM staging system.

Number of invading tumors refers to the number of structures involved by extrathyroidal extension (0 = no ETE; 1-4 = number of involved structures, including muscle, blood vessels, nerves, and trachea/esophageal wall).

### 3.2 LASSO regression screening variables

**Figure 1:**
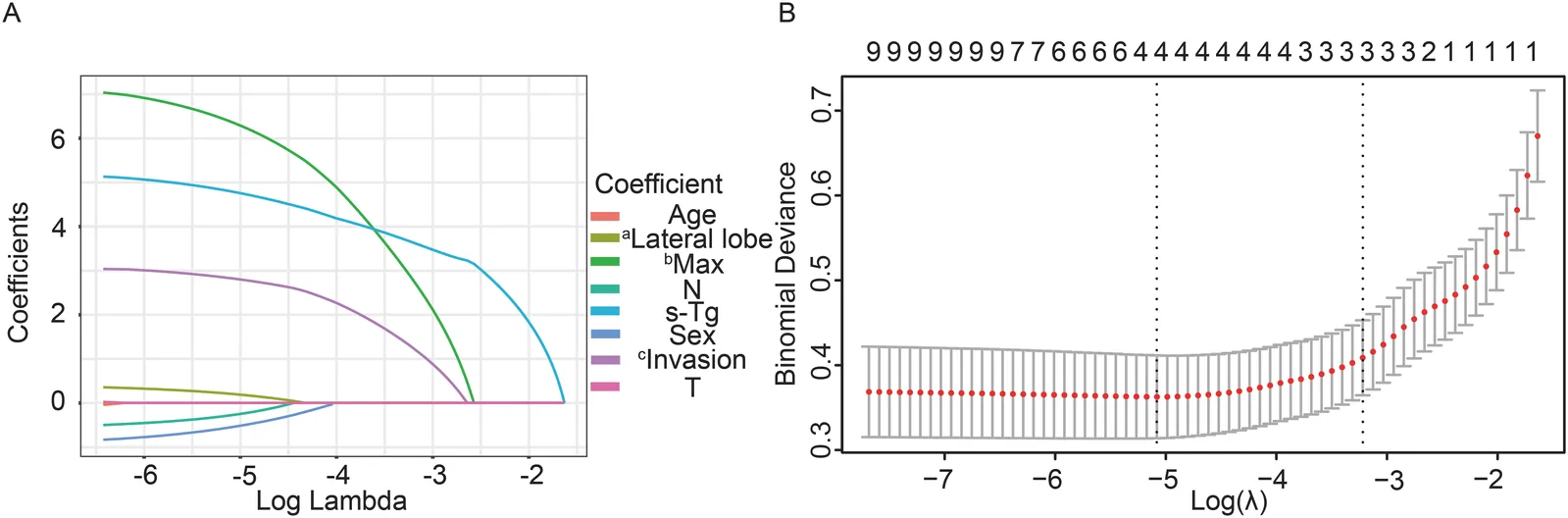
**(A)** LASSO coefficient path graph. **(B)** LASSO regularization path graph via 10-fold cross-validation. The left vertical dashed line indicates lambda.min [log(λ) at which the cross-validation error is minimized]. The right vertical dashed line indicates lambda.1se [the largest log(λ) within one standard error of the minimum]. The numbers at the top of Panel B indicate the number of non-zero coefficients. The model selected lambda.1se to achieve fewer features while maintaining acceptable predictive performance. LASSO, least absolute shrinkage and selection operator. ^a^Lateral lobe: Unilateral or bilateral lobe involvement. ^b^Max: Maximum tumor diameter. ^c^Invasion: Number of invading tumors, which refers to the number of structures involved by extrathyroidal extension (0 = no ETE; 1-4 = number of involved structures, including muscle, blood vessels, nerves, and trachea/esophageal wall).

To avoid the impact of collinearity on the model, we used LASSO regression for feature selection (**Figure 1A**). The optimal penalty parameter λ was determined via 10-fold cross-validation. We selected the largest λ value within one standard error of the minimum cross-validation error (lambda.1se), a standard approach that balances model fit with parsimony (**Figure 1B**). The selected features in the final model included “maximum tumor diameter”, “number of invading tumors”, and “s-Tg”. Among the clinically correlated variables, LASSO preferentially retained maximum tumor diameter over T stage—suggesting that tumor size captured the core prognostic information more directly, while T stage provided little additional predictive value once tumor size was accounted for. Through multivariate binary logistic regression, we confirmed that the three LASSO-selected variables were independent risk factors for lung metastasis (**Table 2**).

**Table 2:** Multivariate binary logistic regression of model variables.

	Coefficient	Standard error	Waldx2	P value	OR (95% CI)
Const	-3.250	0.299	118.125	<0.001	0.039 (0.022-0.070)
Sex	-0.647	0.266	5.900	0.015	0.524 (0.311-0.883)
Age	0.136	0.195	0.489	0.484	1.146 (0.782-1.678)
Unilateral or bilateral lobe involvement^a^	0.037	0.217	0.028	0.866	1.037 (0.678-1.586)
Maximum tumor diameter^a^	0.854	0.212	16.189	<0.001	2.349 (1.549-3.560)
Number of invading tumors^a^	0.572	0.214	7.135	0.008	1.772 (1.165-2.697)
T^b^ stage	-0.093	0.326	0.081	0.776	0.911 (0.481-1.727)
N^b^ stage	0.106	0.230	0.214	0.643	1.112 (0.709-1.745)
s-Tg	1.225	0.174	49.760	<0.001	3.403 (2.421-4.782)

^a^Variables directly extracted from postoperative pathological reports (e.g., Maximum tumor diameter).

^b^Variables derived from postoperative pathology according to TNM staging system.

Number of invading tumors refers to the number of structures involved by extrathyroidal extension (0 = no ETE; 1-4 = number of involved structures, including muscle, blood vessels, nerves, and trachea/esophageal wall).

*p* value < 0.05 was considered statistically significant.

### 3.3 Machine learning model construction and performance evaluation

**Figure 2:**
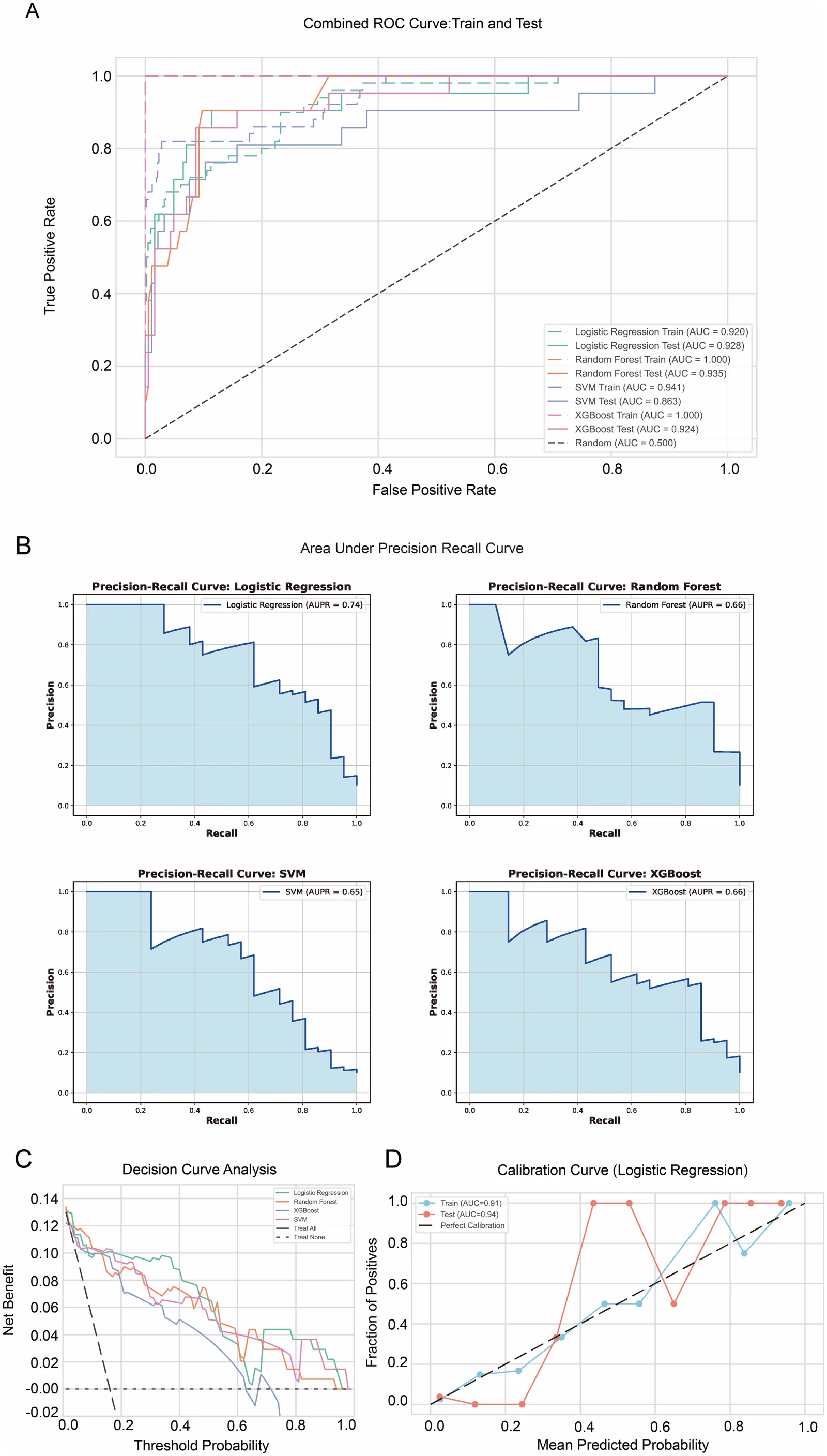
**(A)** ROC curves of the four models in the training and validation sets. **(B)** Precision-recall curves of the four models with area under the precision-recall curve (AUPRC) values. **(C)** Decision curve analysis (DCA) showing the net benefit of the four models at different threshold probabilities. **(D)** Calibration curves of the Logistic Regression model in the training and validation sets. ROC, Receiver operating characteristic; AUC, Area under the curve.

In the training set, we used LASSO-selected feature factors to construct four ML models: LR, RF, SVM, and XGBoost. In the validation set, the area under the curves (AUC)s were 0.928, 0.935, 0.863, and 0.924, respectively. Meanwhile, the area under precision recall curves (AUPRC) were 0.74, 0.66, 0.65 and 0.66, respectively (**Table 3**; **Figures 2A, B**). RF achieved the highest AUC (0.935), while LR showed the highest AUPRC (0.74). Although RF had a marginally higher AUC, LR was selected as the final model because it demonstrated superior performance on imbalanced data (higher AUPRC) and offered better clinical interpretability, enabling the construction of a nomogram for bedside use. The sensitivity and specificity of the LR model were 0.500 and 0.992, respectively, with a positive predictive value of 0.875 and a negative predictive value of 0.946 (**Table 3**). The high specificity (0.992) and positive predictive value (0.875) indicate that when the model predicts a positive result, it is reliable for identifying high-risk patients.

**Table 3:** Performance of each model for prediction.

Model	AUC	Sensitivity	Specificity	PPV	NPV	FPR	FNR	F1 Score	Accuracy
Logistic Regression	0.928	0.500	0.992	0.875	0.946	0.008	0.500	0.636	0.942
Random Forest	0.935	0.429	0.984	0.750	0.938	0.016	0.571	0.545	0.927
XGBoost	0.924	0.357	0.984	0.714	0.931	0.016	0.643	0.476	0.920
Support Vector Machine	0.863	0.286	0.992	0.800	0.924	0.008	0.714	0.421	0.920

AUC, Area under the curve; PPV, Positive Predictive Rate; NPV, Negative Predictive Rate; FPR, False Positive Rate; FNR, False Negative Rate.

**Figure 3:**
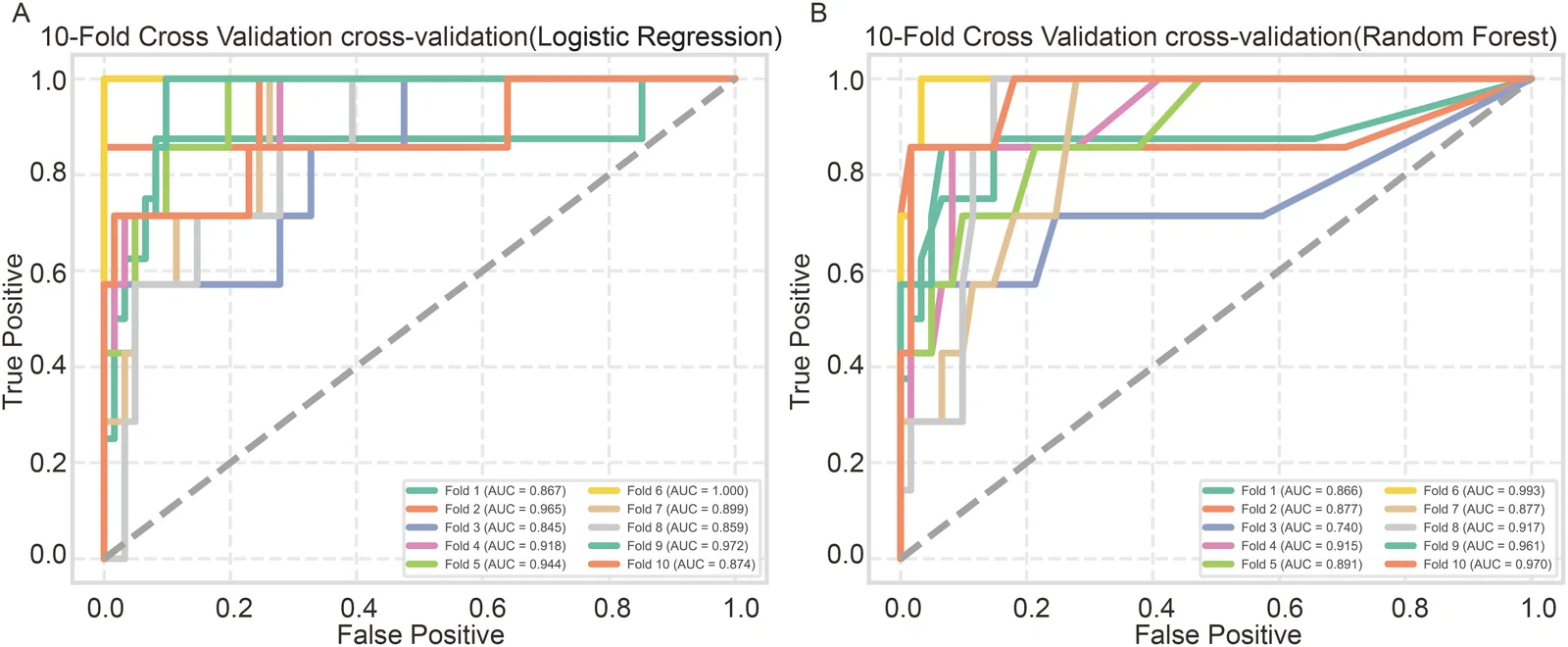
**(A)** 10-fold cross-validation results for the logistic regression model. **(B)** 10-fold cross-validation results for the random forest model.

To compare the net benefits of the four models in clinical decision-making, we performed decision curve analysis (**Figure 2C**). The x-axis represents the threshold probability at which a patient would be considered for intervention, and the y-axis represents the net benefit. The thick dashed line represents the assumption that all patients received intervention (“Treat All”), whereas the thin dashed line indicates that no patients received the intervention (“Treat None”). By stratifying patients based on predicted risk, all four ML-based models provided greater net benefit than either the “Treat All” or “Treat None” strategies across most clinically relevant threshold ranges. At most thresholds, the LR model showed the highest net benefit among the four models. According to the results of 10-fold cross-validation in the training cohort (**Figure 3**), the average AUC value of LR was 0.9143, compared with 0.9007 for RF, further supporting the stability and generalizability of the LR model.

### 3.4 Constructing the LR model calibration curve

The calibration curve of the training set showed good agreement between the predicted probabilities and observed outcomes, indicating that the LR model was well-calibrated in the training set (**Figure 2D**). In the validation set, the calibration curve showed some deviation, though still within an acceptable range.

### 3.5 Building nomogram analysis

**Figure 4:**
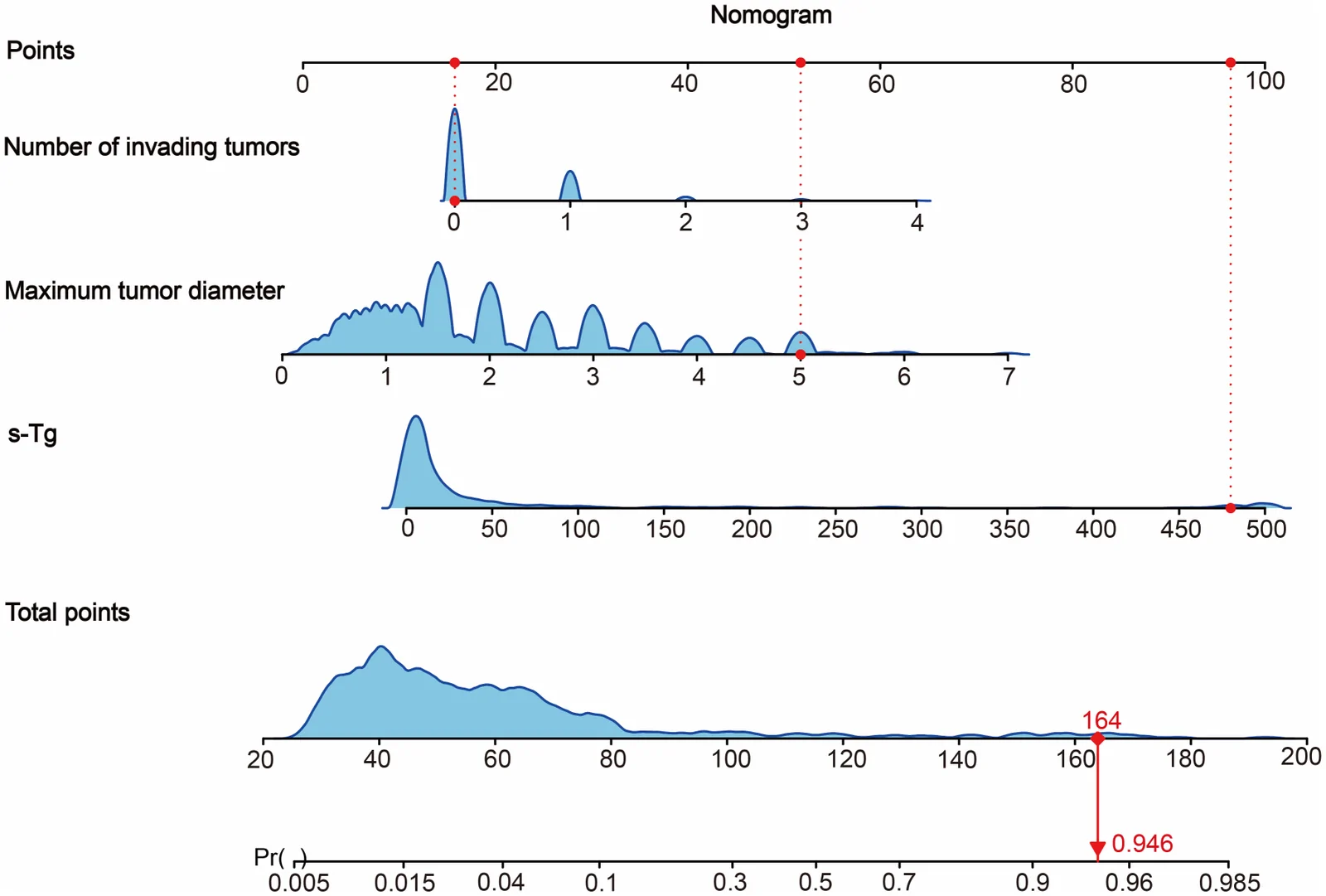
Nomogram predicting lung metastasis in PTC patients based on the Logistic Regression model. PTC, papillary thyroid carcinoma. Number of invading tumors refers to the number of structures involved by extrathyroidal extension (0 = no ETE; 1-4 = number of involved structures, including muscle, blood vessels, nerves, and trachea/esophageal wall).

Based on the final selected LR model, we constructed a nomogram using the training group data (**Figure 4**). The predicted probability of lung metastasis can be obtained by summing the scores assigned to each factor, providing a practical reference for clinical decision-making.

## 4 Discussion

In this retrospective cohort study of 681 PTC patients who received RAI treatment for the first time, LASSO regression identified maximum tumor diameter, number of invading tumors (the number of structures involved by extrathyroidal extension), and s-Tg as the most relevant predictors for lung metastasis. These variables were consistent with previously identified risk factors for lung metastasis in PTC, such as maximum tumor size and extrathyroidal extension, supporting the robustness of our feature selection approach (18–21). Using these LASSO-selected features, we developed and validated four ML models, namely, LR, RF, SVM, and XGBoost, to predict lung metastasis. In the validation set, the AUCs were 0.928, 0.935, 0.863, and 0.924 for LR, RF, SVM, and XGBoost, respectively. Furthermore, the clinical decision curve analysis revealed that the LR model provided greater net benefit than the other three models at most thresholds.

The selection of LR as the final model was primarily justified by its performance on imbalanced data. Although RF achieved a slightly higher AUC (0.935 vs. 0.928), LR demonstrated superior performance on the minority class, as reflected by its higher AUPRC (0.74 vs. 0.66 for RF). In imbalanced datasets, AUPRC is more informative than AUC when the minority class is the primary clinical concern (16). The robustness of the LR model was further supported by 10-fold cross-validation results, which showed a higher average AUC for LR (0.9143) compared with RF (0.9007). In addition, calibration curves also demonstrated acceptable agreement between predicted and observed risks in the validation set, confirming the model’s reliability. LR thus balances predictive performance with clinical interpretability, enabling the construction of an equation-based nomogram that can be readily applied at the bedside (17).

Notably, the LR model showed a relatively low sensitivity of 0.500, indicating that a proportion of patients with lung metastases may be missed. This low sensitivity, however, should be interpreted in the context of class imbalance (71 vs. 610), where the model naturally tends to favor the majority class (non-metastasis) to optimize overall accuracy. Similar findings have been consistently observed across multiple studies in this field. Hou et al. reported RF model sensitivities of 0.447–0.488 in their multicenter PTC cohort, and Su et al. reported a recall rate of 0.64 for their LR model in predicting occult pulmonary metastases—nearly identical to our LR model’s sensitivity (22, 23). Liu et al. also noted that despite high accuracy (0.99), the precision of their RF model was only 0.61 due to extreme class imbalance (24). These converging observations suggest that low sensitivity is not unique to our model but rather reflects an inherent challenge in predicting rare events such as lung metastasis in PTC, where the positive class constitutes only a small fraction of the population.

Some studies have previously developed prediction models for metastasis in thyroid cancer, though with varying scopes and methodologies. Shen et al. developed ML models for lung metastasis risk prediction in DTC based on two databases, demonstrating the feasibility of ML in this context (19). Huang et al. developed an XGBoost model for combined bone/lung metastasis prediction using SEER data with 13,296 patients and applied SMOTE oversampling, achieving a high AUC (0.988/0.866) (25). In contrast to Huang et al., who used SMOTE to address the highly imbalanced SEER data, we chose not to apply resampling techniques in our analysis, as we prioritized model interpretability and addressed class imbalance through appropriate metric selection (AUPRC) and cross-validation. Moreover, unlike models that predict combined bone/lung metastasis (25), our study focused specifically on lung metastasis, which is the most common site of distant metastasis in PTC and therefore has direct clinical relevance. Compared with traditional nomogram-based approaches that rely on logistic regression alone (15, 17), our study systematically compared multiple ML algorithms and selected the model with the best balance of performance and interpretability for clinical use.

Given the high specificity (99.2%) and high positive predictive value (87.5%) of our LR model, its primary clinical utility lies in identifying patients at high risk of lung metastasis who warrant further diagnostic evaluation. When the model predicts a positive result, it provides strong support for considering confirmatory imaging, such as chest CT. Conversely, due to the relatively low sensitivity (50%), a negative prediction should be interpreted with caution and does not reliably exclude the possibility of lung metastasis. Therefore, in clinical practice, the model is best used to guide targeted diagnostic workup by identifying patients who would most benefit from confirmatory imaging. The final treatment decision, including RAI, should remain based on comprehensive clinical evaluation integrating all available clinical, pathological, and biochemical information (6, 8).

## 5 Limitations

This study has some limitations that should be acknowledged. First, it is a retrospective, single-center study with internal validation only, which limits the generalizability of our findings. External validation in independent cohorts, preferably with larger sample sizes, is needed to confirm the model’s robustness across different populations and clinical settings. Second, the dataset was imbalanced (71 vs. 610), which may affect model performance; although we used AUPRC alongside AUC to mitigate this issue, AUPRC should be interpreted in conjunction with other metrics rather than in isolation to avoid overinterpretation. Third, the diagnosis of lung metastasis in this study was confirmed primarily by radiological imaging (CT, SPECT/CT, RxWBS) combined with clinical follow-up and elevated Tg levels. Although pathological confirmation was listed as a diagnostic criterion, it was rarely performed because biopsy of pulmonary metastatic lesions is not routinely conducted in clinical practice. Finally, the factors included in this study were all derived from data that exists for most patients, and we did not include genetic mutations (e.g., BRAF V600E, TERT) or radiomic features, which may further improve prediction accuracy (12). Future multicenter and prospective studies incorporating these additional factors are needed to enhance the model’s robustness and generalizability.

## 6 Conclusion

The LR model demonstrated good predictive performance for lung metastasis in PTC patients who received RAI treatment, with favorable net benefit across most threshold probabilities. The model provides a simple, interpretable risk stratification tool that can support clinical decision-making and facilitate individualized follow-up planning.

## Data Availability

The original contributions presented in the study are included in the article/supplementary material. Further inquiries can be directed to the corresponding author.
